# Supplementary material for: An inter‐species protein–protein interaction network across vast evolutionary distance
Source: Mol Syst Biol. 2016 Apr 23;12(4):865. doi: 10.15252/msb.20156484 (PMC4848758; doi:10.15252/msb.20156484)
Supplement: Supplementary file 1 — Appendix [file MSB-12-865-s001.docx]

Appendix for

**An inter-species protein-protein interaction network across vast evolutionary distance**

Quan Zhong^1,2,3,†,^*, Samuel J. Pevzner^1,2,4,5,†^, Tong Hao^1,2^, Yang Wang^1,2^, Roberto Mosca^6^, Jörg Menche^1,7^, Mikko Taipale^8^, Murat Taşan^1,9,10,11^, Changyu Fan^1,2^, Xinping Yang^1,2^, Patrick Haley^1,2^, Ryan R. Murray^1,2^, Flora Mer^1,2^, Fana Gebreab^1,2^, Stanley Tam^1,2^, Amélie Dricot^1,2^, Patrick Reichert^1,2^, Andrew MacWilliams^1,2^, Balaji Santhanam^1,2^, Lila Ghamsari^1,2^, Michael A. Calderwood^1,2^, Thomas Rolland^1,2^, Benoit Charloteaux^1,2^, Susan Lindquist^8,12,13^, Albert-László Barabási^1,7,14^, David E. Hill^1,2^, Patrick Aloy^6,15^, Michael E. Cusick^1,2^, Yu Xia^1,16,^*, Frederick P. Roth^1,9,10,11,17,^*, & Marc Vidal^1,2,^*

1 Center for Cancer Systems Biology (CCSB) and Department of Cancer Biology, Dana-Farber Cancer Institute, Boston, MA, USA

2 Department of Genetics, Harvard Medical School, Boston, MA, USA

3 Department of Biological Sciences, Wright State University, Dayton, OH, USA

4 Department of Biomedical Engineering, Boston University, Boston, MA, USA

5 Boston University School of Medicine, Boston, MA, USA

6 Joint IRB-BSC-CRG Program in Computational Biology. Institute for Research in Biomecdicine (IRB Barcelona). The Barcelona Institute of Science and Technology. Barcelona, Catalonia, Spain.

7 Center for Complex Network Research (CCNR) and Department of Physics, Northeastern University, Boston, MA, USA

8 Whitehead Institute for Biomedical Research, Cambridge, MA, USA

9 Departments of Molecular Genetics and Computer Science, University of Toronto, Toronto, Ontario, Canada

10 Donnelly Centre, University of Toronto, Toronto, Ontario, Canada

11 Lunenfeld-Tanenbaum Research Institute, Mt. Sinai Hospital, Toronto, Ontario, Canada

12 Department of Biology, Massachusetts Institute of Technology, Cambridge, MA, USA

13 Howard Hughes Medical Institute, Massachusetts Institute of Technology, Cambridge, MA, USA

14 Department of Medicine, Brigham and Women’s Hospital, Harvard Medical School, Boston, MA, USA

15 Institució Catalana de Recerca i Estudis Avançats (ICREA), Barcelona, Spain

16 Department of Bioengineering, McGill University, Montreal, Quebec, Canada

17 Canadian Institute for Advanced Research, Toronto, Ontario, Canada

^†^These authors contributed equally to this work

*Corresponding author. Tel: 1 617 632 5180; E-mail: marc_vidal@dfci.harvard.edu

Corresponding author. Tel: 1 416 946 5130; E-mail: fritz.roth@utoronto.ca

Corresponding author. Tel: 1 514 398 5026; E-mail: brandon.xia@mcgill.ca

Corresponding author. Tel: 1 937 775 3571; E-mail: quan.zhong@wright.edu

**Running title:** A human-yeast inter-species interactome

**Appendix Supplementary Methods**

**A. Inter-interactome mapping and validation strategies**

**Yeast strains:** The yeast strains used were Y8800 and Y8930, of mating type *MAT*a and *MAT*α respectively (Dreze et al, 2010). Ability to activate the *GAL1*-*HIS3* reporter gene was determined by growth on synthetic medium lacking histidine and supplemented with 1 mM 3-AT (3-amino-1,2,4-triazole, a competitive inhibitor of the *HIS3* gene product).

**Search space:** The inter-species search space is the 2-dimensional combinatorial space of the 7,240 AD-ORF hybrid constructs of the human ORFs contained in the hORFeome1.1 resource (Rual et al, 2004) and the DB-ORF hybrid constructs of 3,778 non-autoactivator yeast ORFs (Yu et al, 2008).

**Mapping inter-species interactions of human proteins that complement yeast proteins:** We obtained from the *Saccharomyces* Genome Database (SGD) (Cherry et al, 2012) (http://www.yeastgenome.org/) a list of publications describing studies involving cross-species gene expression in *S. cerevisiae*. We manually selected papers that described functional complementation experiments between human and yeast. We identified human orthologs of yeast genes that were described in each selected paper as provided by SGD. There were a total of 172 such human genes available as cloned ORFs in our ORFeome collection (Lamesch et al, 2004). We pooled the AD-ORF constructs encoding these 172 human proteins as one pool and carried out Y2H screens against DB-ORF constructs of 3,778 non-autoactivator yeast ORFs. The Y2H first pass, pairwise test and confirmation procedures are the same as described below for the systematic screen. We identified 46 inter-species interactions involving 15 human proteins that functionally complemented their corresponding orthologs in yeast (Table EV1). Comparison of the identified inter-species interactions to intra-species yeast interactions is described in Materials and Methods.

**Systematic Y2H screen:** We systematically mated on solid rich medium (YEPD) sets of 94 individual *MAT*α Y8930 DB-X yeast strains, arrayed in a 96-well format, with *MAT*a Y8800 AD-Y mini-libraries. Each 96-well plate of DB-X yeast strains was mated against each of 48 AD-Y mini-libraries, each mini-library containing 192 AD-Y yeast strains. After overnight incubation at 30°C, yeast cells were transferred onto synthetic media lacking histidine and supplemented with 1 mM 3-AT (-His media). Diploids that could grow under this selective condition indicate activation of the *GAL1*-*HIS3* Y2H reporter gene (His+ phenotype). In parallel yeast cells were also transferred onto DB-auto-activator detection media (-His + 1mg/l cycloheximide (CHX), hereafter -HisCHX) Growth on this media constitutes a His+CHX^R^ phenotype. The pDEST-AD-*CYH2* vector carries the *CYH2* counter-selectable marker, which allows for plasmid shuffling on CHX containing media. This control step identifies auto-activators that spontaneously arise during the Y2H selection process. Auto-activating DB-X yeast strains show a His+ / His+CHX^R^ phenotype, whereas genuinely interacting positives show a His+ / His+CHX^S^ phenotype.

Since each DB-X yeast strain is mated against a mini-library of 192 AD-Y yeast strains, it is possible, albeit unlikely, to obtain multiple interactions per mini-library. To mitigate this event we picked four colonies (primary positives) per growth spot. In total ~15,700 primary positive colonies that exhibited a His+ / His+CHX^S^ phenotype were picked from -His plates into a second-generation set of 96-well plates. The primary positive colonies were retested for Y2H reporter activation and auto-activation with both Y2H reporter genes (*GAL1*-*HIS3* and *GAL2*-*ADE2*). Nearly 11,500 primary positive colonies activated at least one reporter gene and were CHX sensitive. These secondary positives were retained for further processing.

**Identification of interaction sequence tags (ISTs):** PCR stitching followed by massively parallel 454 FLX sequencing was used to identify the ORFs encoding interacting proteins in the secondary positives pairs. For the PCR reactions, yeast cells from positive colonies are lysed in 15 μl of lysis buffer (2.5 mg/ml zymolase 20T (21,100 U/g; Seikagaku) dissolved in 0.1 M sodium phosphate buffer (pH 7.4)) in each well of a 96-well PCR plate. From each positive well a small amount of yeast cells (not more than what fit on the end of a standard 200-μl tip) is picked and resuspended in lysis buffer in soft-shell, V-bottom 96-well microtiter plate (hereafter called PCR plate). PCR plates are placed in a thermocycler and subjected to the following program: 37°C for 15 min, 95 °C for 5 min and hold at 10°C. To each well 100 μl of filter-sterilized water are added, then PCR plates are centrifuged for 10 min at 800 g and stored at −20°C. PCR amplifications of DB-X and AD-Y were carried out using Platinum HiFi polymerase (Invitrogen). The primers used to amplify DB-X are:

5’-GGCTTCAGTGGAGACTGATATGCCTC-3’ (DB-primer)

5’-CTCTCAGCTCGGCGGTATCCCCATCAAACCACTTTGTACAAGAAAGTTGGG-3’.

The primers used to amply AD-Y are

5’-CGCGTTTGGAATCACTACAGGG-3’ (AD-primer)

5’-GGATACCGCCGAGCTGAGAGCCATCAAACCACTTTGTACAAGAAAGTTGGG-3’. The stitching PCR of DB-X and AD-Y was carried out using KOD Hot Start DNA polymerase (Novagen) and the DB- and AD-primers. From each well of a 96 well PCR plate, 5 μl aliquots of each stitched PCR product are removed and all aliquots are combined together. A 1 ml aliquot of the pooled stitched PCR products is purified using a QIAquick PCR Purification kit (Qiagen). A 200-μl aliquot of the purified stitched PCR products is used for 454 FLX sequencing.

For 454 FLX sequencing, PCR products are processed using kits supplied by the manufacturer: GS Standard DNA Library Preparation kit; GS FLX Standard emPCR kit (Shotgun); GS FLX PicoTiterPlate kit (70 × 75); and GS FLX Standard LR70 Sequencing kit. A 3-5 μg sample of the pooled PCR products is fragmented by nebulization for 1 min under nitrogen gas pressure of 30 p.s.i. (2.1 bar), then the DNA fragments are size-selected and subjected to end-polishing and adaptor ligation. The DNA fragment library, after being immobilized on streptavidin-coated beads, is subjected to a PCR fill-in reaction to repair the gaps generated by the ligation of non-phosphorylated adaptors to the fragments. The single-stranded library is obtained by melting off the non-biotinylated strand of bead-bound fragments. Subsequent quality assessment and quantification are done by 96-well plate fluorometry and analysis on a Bioanalyzer with Agilent RNA Pico 6000 LabChip kit. The amount of library DNA needed for optimal results in the emulsion-based clonal amplification (emulsion PCR) procedure is determined by emulsion titration assay according to instructions supplied by the manufacturer. The library of DNA fragments is amplified from a single bead-bound copy to millions of copies per bead by water-in-oil emulsion PCR. Emulsions are broken and the beads carrying the amplified library are recovered with biotinylated amplification primers and streptavidin-coated magnetic beads following protocols provided by the manufacturer. Beads are counted, the enrichment ratio is calculated and the recommended amount of sequencing primer is added to bead-bound amplified fragments. After annealing and mixing of DNA-loaded beads with packing beads, the wells of a GS FLX Standard PicoTiterPlate are loaded according to protocols supplied by the manufacturer. The loaded PicoTiterPlate is inserted into the 454 FLX instrument and run according to the standard protocol.

From the 454 FLX sequencing data, we first identified all usable sequencing reads containing the 82-bp linker using the 'cross_match' protocol. Then DB-X and AD-Y were identified by mapping both ends of usable sequencing reads to the screened ~6,000 ORFs in yeast ORFeome and to the ~8,000 ORFs in human ORFeome 1.1 and by BLASTN (mismatches allowed) with an E-value cutoff of 10^−3^. The quality of obtained ISTs is evaluated by moving a sliding window of 20 nucleotides to define portions of ISTs with an average PHRED score greater than or equal to 30 over at least 10% of their lengths. Unique IST pairs with a BLASTN E-value less than or equal to 10^-3^ are retained. When an IST could not be unambiguously assigned to a single ORF, because multiple ORF isoforms correspond to the same locus, we provisionally assigned the IST to all possible ORF matches, ultimately only keeping those that passed the subsequent verification step.

**Y2H verification:** The interaction phenotype of all candidate Y2H interaction pairs is experimentally verified individually, to ensure reproducibility and to exclude the chance that physiologic and genetic changes occurring during the course of the screen have given rise to experimental artifacts. We verified the Y2H phenotype of candidate Y2H pairs by mating on YEPD media the matching individual *MAT*α Y8930 DB-X yeast strains and *MAT*a Y8800 AD-Y yeast strains. We selected diploid cells on solid SC-Leu-Trp selective media and tested them for activation of both the *GAL1*-*HIS3* and *GAL2*-*ADE2* reporter genes. To control for technical variability and to increase the reproducibility, all pairs were re-tested four times independently by four separate experimenters. Only pairs that gave rise to a His+ and a HisCHX- growth phenotype in four out of four replicates, the highest stringency, were considered verified. Of the 2,836 pairs tested, 1,671 (59%) scored positive according to these criteria. All interactions that were reproduced in four replicates are considered verified Y2H interactions and were included in the **y**east-**h**uman inter-species **i**nter-**i**nteractome (YHII-1) data set.

**Y2H confirmation:** In the last step of our Y2H screening protocol, the phenotypes of each verified Y2H interaction pair are tested once more on selective -His and -HisCHX plates. We also assess each individual hybrid construct (DB-X and AD-Y) separately for possible spontaneous auto-activation. Only those pairs whose phenotype could be confirmed, and whose respective hybrid constructs are not auto-activators, are retained for identity confirmation by end-read sequencing of DB-X and AD-Y PCR products amplified directly from yeast cells. Of the 1,671 verified Y2H interacting pairs, the phenotype and identities of 1,583 (95%) pairs were confirmed (Table EV4).

**B. Comparisons of inter-species and intra-species interactions by overlapping with protein structural complexes**

**Enrichment calculation:** We found a total of 17 of inter-species interactions for which the yeast or human orthologs can be mapped in the same structural protein complexes (Table EV4) nine in yeast complexes and 12 in human complexes, with an overlap of four found in interologous protein complexes in both organisms. Log_2_ enrichment was calculated as follows:

| All human–yeast protein pairs in the inter-interactome mapping space, in which either has an ortholog in the opposing species | | The human–yeast protein pairs corresponding to intra-species protein pairs in the same 3D complexes | |
| --- | --- | --- | --- |
|  |  | Yes | No |
| Identified in the inter-interactome | Yes | 17 | 1,563 |
|  | No | 1,754 | 14,311,505 |

17/(17+1,754)

Enrichment = log_2_ [ ] = 6.46

1,563/(1,563+14,311,505)

*P* < 2 × 10^-27^ as calculated by Fisher’s Exact test.

Of each of the 17 inter-species interacting pairs, the yeast or human ortholog and the inter-species interactor were found to be in direct contact in the structural model. This is statistically significant (*P* < 1 × 10^-6^ as calculated by Fisher’s Exact test):

| All human–yeast protein pairs corresponding to intra-species protein pairs in the same 3D complexes | | The human–yeast protein pairs corresponding to intra-species protein pairs in the same 3D complexes with direct residue-residue contact | |
| --- | --- | --- | --- |
|  |  | Yes | No |
| Identified in the inter-interactome | Yes | 17 | 0 |
|  | No | 792 | 962 |

Thus, inter-species interactions originating from ancestral binding sites significantly overlap with intra-species interactions, supported by direct residue-residue contacts in 3D structures.

**References**

Cherry JM, Hong EL, Amundsen C, Balakrishnan R, Binkley G, Chan ET, Christie KR, Costanzo MC, Dwight SS, Engel SR, Fisk DG, Hirschman JE, Hitz BC, Karra K, Krieger CJ, Miyasato SR, Nash RS, Park J, Skrzypek MS, Simison M et al (2012) Saccharomyces Genome Database: the genomics resource of budding yeast. *Nucleic Acids Res* **40:** D700-705

Dreze M, Monachello D, Lurin C, Cusick ME, Hill DE, Vidal M, Braun P (2010) High-quality binary interactome mapping. *Methods Enzymol* **470:** 281-315

Lamesch P, Milstein S, Hao T, Rosenberg J, Li N, Sequerra R, Bosak S, Doucette-Stamm L, Vandenhaute J, Hill DE, Vidal M (2004) *C. elegans* ORFeome version 3.1: increasing the coverage of ORFeome resources with improved gene predictions. *Genome Res* **14:** 2064-2069

Rual JF, Hirozane-Kishikawa T, Hao T, Bertin N, Li S, Dricot A, Li N, Rosenberg J, Lamesch P, Vidalain PO, Clingingsmith TR, Hartley JL, Esposito D, Cheo D, Moore T, Simmons B, Sequerra R, Bosak S, Doucette-Stamm L, Le Peuch C et al (2004) Human ORFeome version 1.1: a platform for reverse proteomics. *Genome Res* **14:** 2128-2135

Yu H, Braun P, Yildirim MA, Lemmens I, Venkatesan K, Sahalie J, Hirozane-Kishikawa T, Gebreab F, Li N, Simonis N, Hao T, Rual JF, Dricot A, Vazquez A, Murray RR, Simon C, Tardivo L, Tam S, Svrzikapa N, Fan C et al (2008) High-quality binary protein interaction map of the yeast interactome network. *Science* **322:** 104-110
