# Supplementary material for: An inter‐species protein–protein interaction network across vast evolutionary distance
Source: Mol Syst Biol. 2016 Apr 23;12(4):865. doi: 10.15252/msb.20156484 (PMC4848758; doi:10.15252/msb.20156484)
Supplement: Supplementary file 2 — Expanded View Figures PDF [file MSB-12-865-s002.pdf]

Expanded View Figures

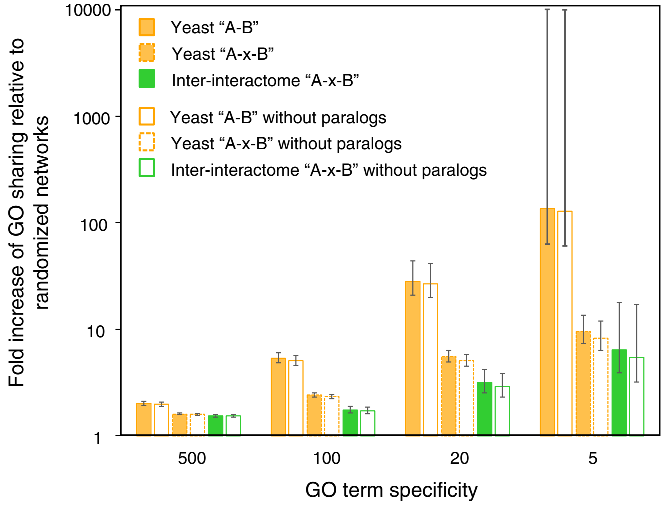

Figure EV1. Enrichment of GO annotation upon removal of paralogs.
Fold increase of interacting proteins sharing specific GO in the inter-interactome (YHII-1, green) and intra-species yeast (YI-1) networks before and after removal of paralogs relative to 1,000 randomized network controls at indicated four cutoffs of GO specificity (*x*-axis). Error bars indicate standard errors. Empirical *P*-values for all cases are 0.001.

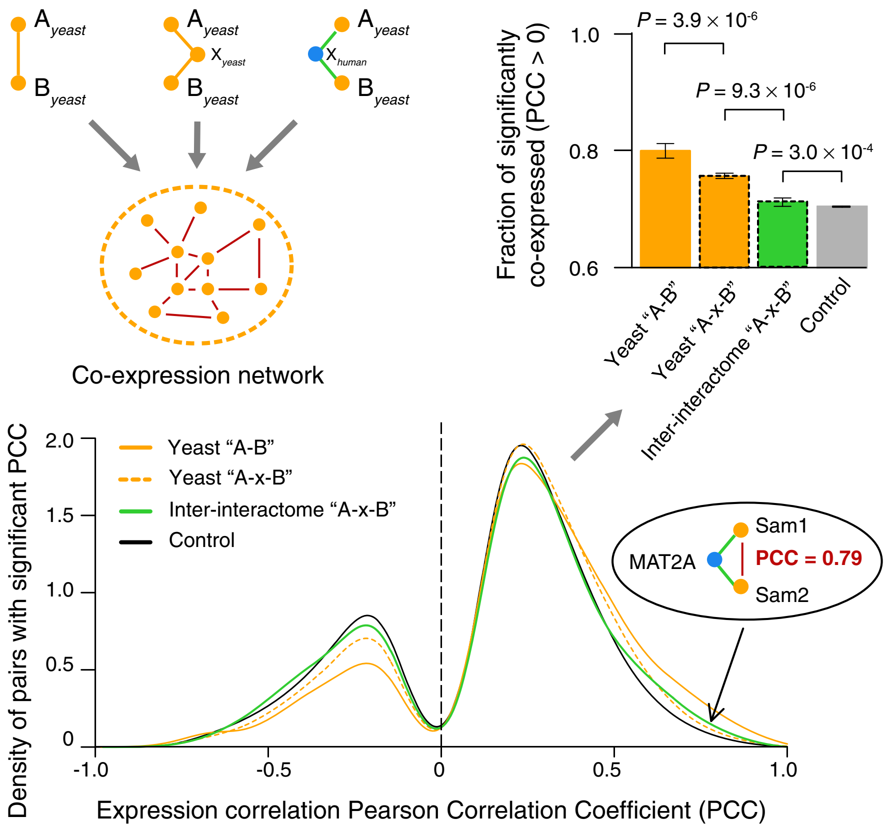

Figure EV2. Overlap with gene expression correlation network.
Distribution of co-expression PCCs for directly and indirectly interacting protein pairs. Fraction of each distribution with PCC > 0 (top right). Error bars: standard error of the proportion. *P*-values for PCC distribution comparisons determined by Mann–Whitney *U*-test. An inter-species example pair is shown.

© 2016 The Authors
